# Efficient Quasi-Resonant, Polarization-Selective Excitation of GaN Quantum Emitters

**DOI:** 10.1021/acsphotonics.6c02170

**Published:** 2026-10-15

**Authors:** Nilesh Dalla, Paweł Kulboka, Michał Kobecki, Jan Misiak, Paweł Prystawko, Tomasz Kazimierczuk, Piotr Kossacki, Henryk Turski, Tomasz Jakubczyk

**Affiliations:** † 49605 University of Warsaw, Faculty of Physics, Institute of Experimental Physics, Ludwika Pasteura 5, Warsaw 02-093, Poland; ‡ Institute of High Pressure Physics “Unipress”, Polish Academy of Sciences, Sokołowska 29/37, Warsaw 01-142, Poland

**Keywords:** GaN Defects, Single Photon Emitter, Photoluminescence Spectroscopy

## Abstract

Defect centers in gallium nitride (GaN) emerge as bright sources of single photons, which recently have been demonstrated to optically interface a localized spin. However, the structure and composition of these defects as well as their efficient excitation techniques have not been a subject of thorough studies. This work presents evidence that by tuning the excitation laser energy to specific resonance values, the excitation efficiency can be enhanced, resulting in a relative increase of photoluminescence intensity by up to an order of magnitude. The resonances can be selectively addressed with linearly polarized light, while the emission dipoles remain unchanged, enabling polarization-controlled enhancement. These results establish an efficient method of excitation for GaN-based emitters, thereby increasing the generation rate of photons. The data are consistent with excitation via localized vibrational modes (LVMs) associated with point-defect complexes, establishing a practical quasi-resonant route to brighter, polarization-addressable operation of GaN defect emitters and clarifying their energy-level structure.

## Graphical abstract

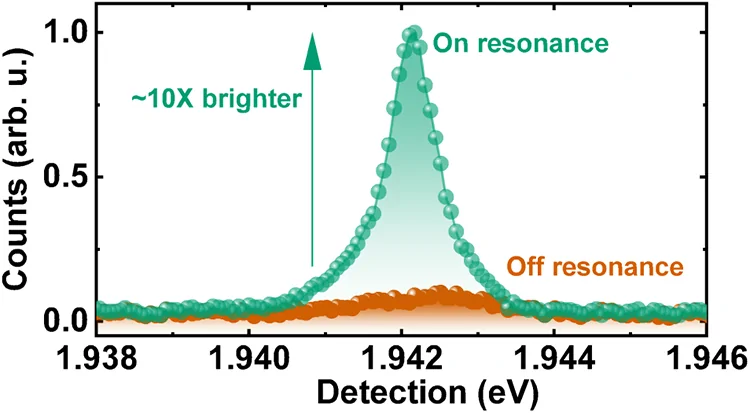

## Introduction

Gallium nitride (GaN) is widely recognized for its diverse applications in high-speed electronics, high breakdown-voltage devices, and blue light-emitting diodes, owing to its wide band gap.^1^ While structural defects are generally detrimental to high-performance electronic devices, the optical properties of GaN defects have attracted considerable scientific interest. In particular, defect-related single-photon emission in GaN has emerged as a promising platform for quantum-photonics applications. GaN hosts single-photon emitters (SPEs) that operate across a broad spectral range, from near-ultraviolet^2^ to near-infrared,^3,4^ offering versatility for quantum communication technologies. These defects have been shown to exhibit bright, stable single-photon emission even at room temperature.^5^ Recent studies also indicate the existence of a spin degree of freedom associated with visible and IR range defects.^6,7^ The recent demonstration of controlled epitaxy of room-temperature quantum emitters in GaN^8^ marks an important step toward scalable quantum photonic technologies based on defect engineering. The emission characteristics are strongly influenced by the defect type, spatial distribution, and substrate selection. Although near-infrared defects are particularly attractive due to reduced optical losses^9^ in fibers, practical implementations of telecom-wavelength single-photon emitters remain limited.^10,11^ This has been partly attributed to more prominent nonradiative processes in the infrared regime, which reduce the overall emission efficiency.^12^ These characteristics render visible-range emitters particularly interesting from a scientific perspective. A comprehensive understanding of the origin and energy-level structure of these defects is essential to fully exploiting their potential; however, both remain largely unresolved.^13^ Early studies suggested that such defects could arise from crystal dislocations or stacking faults.^14^ More recent investigations, however, indicate that the emitters may instead originate from substitutional atoms or complex combinations of substitutional defects.^15^

Carbon is one of the most common unintentional dopants in GaN. It is known to introduce deep level defects leading to localized electronic and vibrational levels. Hybrid functional DFT and experimental studies have shown that carbon substituting nitrogen or gallium cites (C_N_ or C_Ga_) can give rise to visible-range luminescence and distinct local vibrational modes.^16–19^

One of the other promising candidates is the N_Ga_V_N_^0^ complex, which is predicted to exhibit a zero-phonon line (ZPL) in the 600–700 nm range and an excited-state lifetime of approximately 3.5 ns,^20^ consistent with experimental observations. Nevertheless, further research is required to definitively determine the microscopic nature and energy-level structure of these visible-range defect emitters.

This work addresses how visible GaN quantum emitters respond to excitation at different photon energies, whether the corresponding excitation pathways exhibit polarization and temperature dependencies, and what their characteristic energy scales reveal about the underlying excitation mechanism.

## Results and Discussion

### Line Width and Autocorrelation

We first establish baseline optical properties and the single-photon character of the emitters used in the subsequent excitation spectroscopy measurements.

The sample surface was characterized at room temperature using a custom-built confocal microscope. Samples exhibiting suitable emitter distributions were subsequently examined at cryogenic temperatures (10 K) by mounting the sample in a cryostat. The emitters were reidentified by scanning the excitation beam across the field of view using a solid immersion objective^21^ or using an aspheric lens mounted on attocube piezo positioners. Quasi-resonant excitation at 2.13 eV (580 nm) was provided by a rhodamine-based laser, and the emission was spectrally isolated from the excitation using appropriate optical filters before detection with a spectrometer. The excitation power was maintained at 1 mW. One such exemplary emitter, labeled as A0, is shown in Figure 1A. The measured full width at half-maximum (FWHM) of the emission line was extracted to be 374 μeV, consistent with values obtained from the previously studied samples.^22^ In our experiments, we also studied samples with varying carbon concentration; however, we have not obtained any conclusive results of impact of carbon concentration on emitter density.

**Figure 1:**
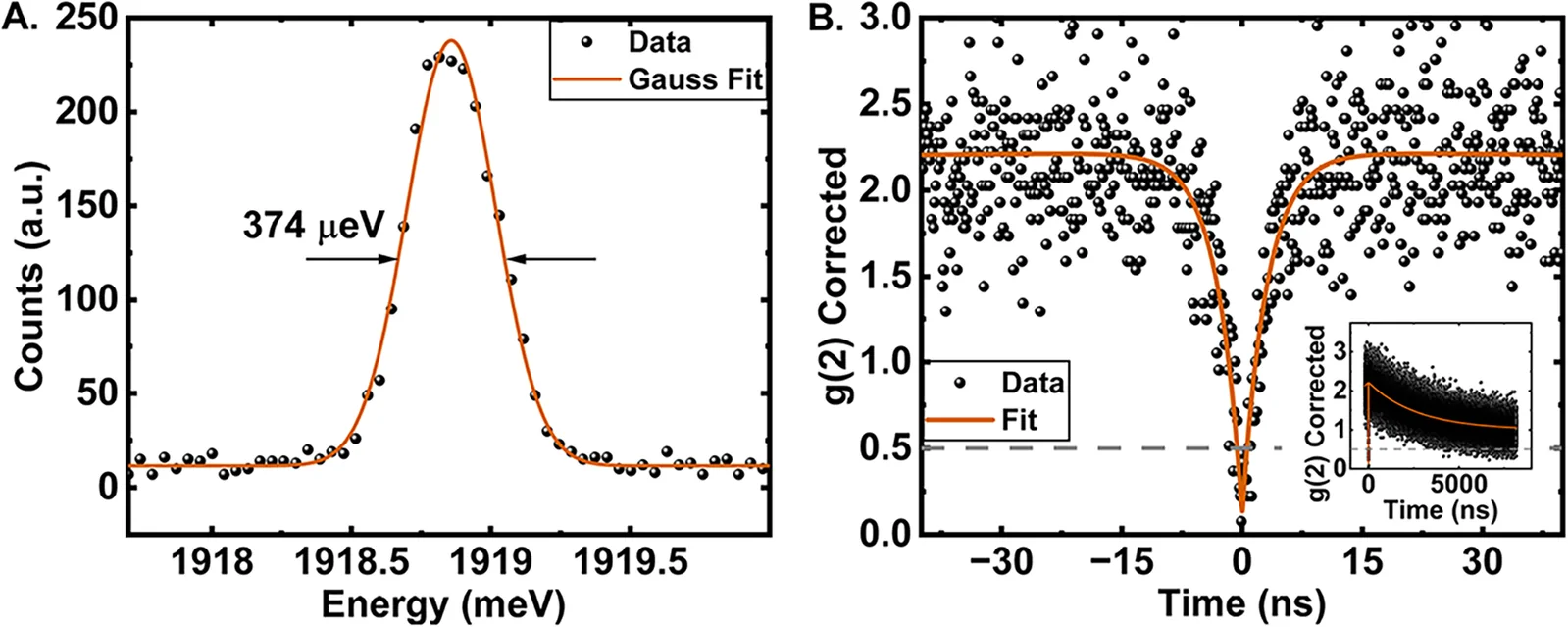
(A) Photoluminescence signal from a single emitter A0. The excitation power was kept at 1 mW and excitation energy was 2.13 eV (580 nm). Gaussian fit shows a (FWHM) of 374 μeV. (B) Corrected second-order correlation count measurement of the same emitter showing in the black color. In the orange color, the fitted curve with *g* ^(2)^(0) = 0.11 ± 0.08 confirming single-photon emission and fitted values for fast and slow decays as *t*_fast_ = 3. 08 ± 0.16 ns and *t*_slow_ = 2698 ± 11 ns. The inset shows the long-delay component of the emitter.

For the same emitter A0, the background-corrected counts *g*_corrected_^(2)^ are shown in Figure 1B. The background-corrected autocorrelation data were fitted using a three-level model (see Supporting Information for more information about fit and background correction). The resulting fit, shown in Figure 1B (orange curve), reveals two distinct dynamical components: a fast decay process with characteristic time *t*_fast_ occurring near zero delay, and a slower component with characteristic time *t*_slow_ at longer delays. The pronounced bunching observed around zero delay can be attributed to population trapping in a metastable shelving state. From the fit, we obtain *t*_fast_ = 3. 08 ± 0.16 ns and *t*_slow_ = 2698 ± 11 ns. The extracted value at zero delay is *g* ^(2)^(0) = 0.11 ± 0.08. Note the error bars here refer to the standard error. The *g* ^(2)^(0) is well below the classical limit of 0.5, thereby confirming the single-photon nature of the emission. See Supporting Information for more correlation results.

### Photoluminescence Excitation (PLE) Scans

We next investigate whether GaN quantum emitters can be excited efficiently through discrete states above the zero-phonon line and whether such excitation provides an advantage over nonresonant pumping. To do so, we conducted a detailed photoluminescence excitation (PLE) study. The excitation energy was scanned over a coarse range from 2.40 to 1.90 eV (520–650 nm) using a supercontinuum source, and over a finer range from 2.17 to 2.10 eV (570–590 nm) using a Rhodamine laser. For each excitation energy, the detection energy range was fixed. The excitation power and sample temperature were maintained at the same values as described earlier. The zero-phonon lines (ZPLs) of three representative emitters are shown in Figure 2A, where the labeling of emitters A0, B, C, E, and so on follows the convention established in our previous work^22^ (the same labels refer to the same emitters in both works). The PLE amplitude was extracted by fitting Gaussian functions to the PLE spectra and is presented in Figure 2B. Emitters B and C exhibit a coarse dependence of the excitation efficiency on the excitation energy, with distinct peaks appearing in the 2.00–2.10 eV range. A fine-resolution scan of emitter E within 2.00–2.15 eV spectral window resolved a set of narrow resonances.

**Figure 2:**
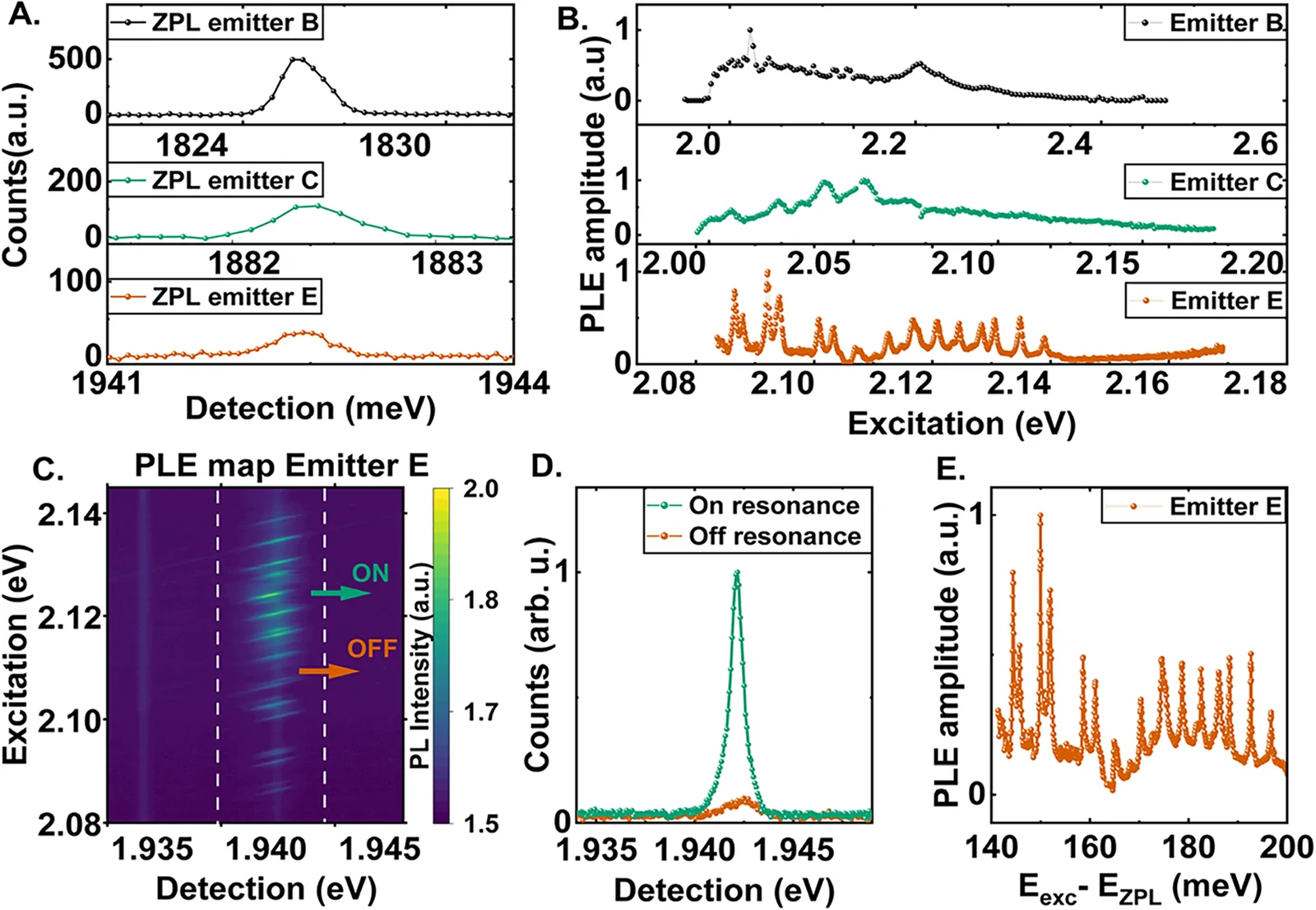
(A) ZPL of three shown emitters, namely, B, C, and E. (B) PLE amplitude plotted for the corresponding B, C, and E emitters. With emitter E showing strong distinct peaks. (C) PLE map of Emitter E with ZPL at 1.942 eV. On and Off arrows represent cross-sections with excitation laser matching or not matching resonance energies. (D) On and Off cross-section spectra of the PLE map. (E) Gaussian fit amplitude of the ZPL (PLE map emitter E) plotted relative to the excitation energy. The distinct peaks correspond to resonance-energy values at discrete energy offsets from the ZPL.

For roughly 80% of the measured emitters, we observed a pronounced increase in emission intensity as the excitation laser was tuned through specific energies. This enhancement is evident in the PLE map of emitter E, presented in Figure 2C. When the excitation energy is resonant with discrete excited states of the emitter, sharp and narrow spectral features emerge, intersecting the zero-phonon line (ZPL). Such narrow resonances are not universal across all emitters and, in selected cases, appear significantly weaker. Representative cross-sections of the PLE map, corresponding to on-resonance and off-resonance excitations (marked “On” and “Off”, respectively), are shown in Figure 2D. Excitation via the strongest resonance results in about an order of magnitude enhancement of the ZPL emission.

To quantify the detuning of the narrow resonances from the ZPL, a subsection of the PLE map (indicated between white dashed lines in Figure 2C) was analyzed, and PLE amplitude fit is shown in Figure 2E. The data reveal that resonances occur at discrete energy offsets from the ZPL. Furthermore, the brightness of these resonances varies, with some exhibiting a significantly higher intensity than others. Finally, the line width of the resonances was measured to be approximately 0.5 meV.

### Polarization-Resolved PLE Study

To determine whether the excitation resonances reflect a structured optical transition rather than an isotropic absorption background, we examine their dependence on excitation polarization relative to the GaN crystallographic axes. For excitation polarization control, the laser beam was passed through a linear polarizer followed by a half-wave (λ/2) plate. Then, for each excitation angle, the excitation energy was swept. Similarly, to characterize the emission polarization, a half-wave plate and a linear polarizer were placed in the detection path before the spectrometer. In order to record angle-resolved PLE plots for each emitter, the emission signal was collected through a monochromator on an avalanche photodiode unit. All of the angles measured as shown in Figure 3 are measured from the GaN in-plane m-direction ([1–100]). All of the collected spectra have been power-normalized since we operate in a linear power dependence regime (see Figure S3 in the Supporting Information). Then onward, for a given excitation energy, the excitation/detection polarization was rotated and resulting spectrum was recorded on the spectrometer. The recorded spectrum was then fitted with Gaussian peak and the extracted amplitude was recorded and plotted to show the dipole pattern of the emitter in absorption or detection. All of the dipoles were then fitted with *A* + *B*·cos^2^ (ϕ + ϕ_0_) to deduce the dipole angle (ϕ_0_). In this experiment, we studied 44 different emitters in this manner, and we find two such group of emitters explained below.

**Figure 3:**
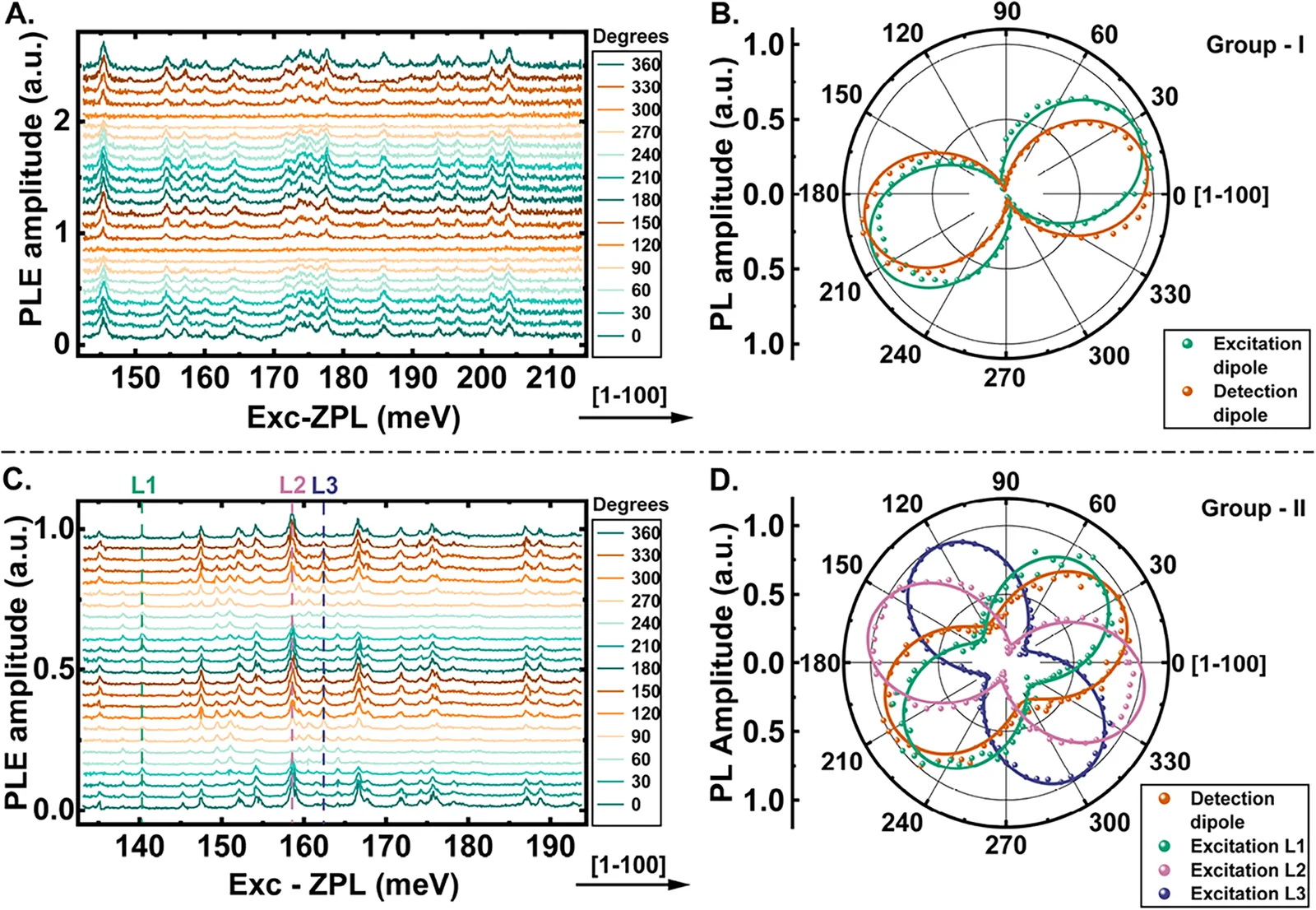
(A) Angle-resolved PLE spectra of emitter F, with the resonance energies shown relative to the ZPL. All angles are referenced from in-plane [1–100] crystallographic direction (m-direction) of GaN. (B) Emission and excitation/absorption dipole pattern of emitter F. Solid lines correspond to the fit for dipole data. (C) Angle-resolved PLE spectra of emitter G, with the resonance energies shown relative to the ZPL. The labels L1, L2, and L3 indicate three distinct resonance energies. (D) Emission dipole pattern and absorption dipole patterns measured for emitter G at the three resonance energies, L1, L2, and L3, as indicated in panel (C).

Angle-resolved PLE spectra of an emitter F have been shown in the Figure 3A. It can be seen that a number of peaks appear in the PLE spectra of the emitter when the excitation is kept at 0°. As the excitation polarization direction is tuned, all of the resonances disappear together at about 120°. From this point onward, all of the resonances appear together and reach a maximum at about 195°. Therefore, for this emitter, all of the resonances are polarized in the same direction. Figure 3B shows the recorded emission pattern and the absorption dipole pattern of emitter F, shown with circles. The line plot shows the fit for a given dipole data set. The absorption pattern lies in agreement with the full-angle-resolved PLE plot. This is the most commonly observed behavior of the emitters that we studied. Such emitters that have the resonances polarized in the same direction, we classify as group 1 emitters.

Another example of a different emitter, emitter G, is shown in Figure 3C,D. The polarization-resolved PLE is shown in Figure 3C. At first glance, many resonance peaks are present for the given emitter. However, on a careful inspection, it shows that there are three different groups of peaks in this plot. Each peak in this plot falls into one of the three groups. Each peak group behaves slightly differently when the polarization direction changes. One such line of each group is represented by L1, L2, and L3 on the plot. The corresponding absorption dipole patterns for each line L1–L3 are plotted separately in Figure 3D and marked with same colors correspondingly. We observe that there are 3 different absorption dipole orientations that this emitter can have depending on the excitation energy. Emission dipole pattern of the emitter has been also plotted in Figure 3D. The emission dipoles remain the same regardless of the excitation energy. Such emitters are classified as group 2 emitters, where the absorption dipole orientation is no longer unique and depends on the excitation energy. We do not observe any correlation between emitter group (1 or 2) and excitation energy, emission energy, or emission dipole orientation relative to the GaN high-symmetry axes (see Figure S6 in the Supporting Information).

### Temperature and Excitation Energy Dependence of the Resonances

We investigate whether the resonance energies remain fixed relative to the ZPL as the temperature changes, which provides a test of their relationship to the internal energy structure of the emitter. PLE scans were thus repeated at three different temperatures for emitter H. The extracted resonance positions relative to the excitation energy are presented in Figure 4A, with the inset providing a magnified view of the spectral region near 144 meV. The data show that the resonance positions remain unchanged with temperature. However, the overall emission intensity decreases with an increase in temperature, accompanied by a slight broadening of the resonance peaks. This behavior may indicate a phonon-mediated contribution to the observed resonance.

**Figure 4:**
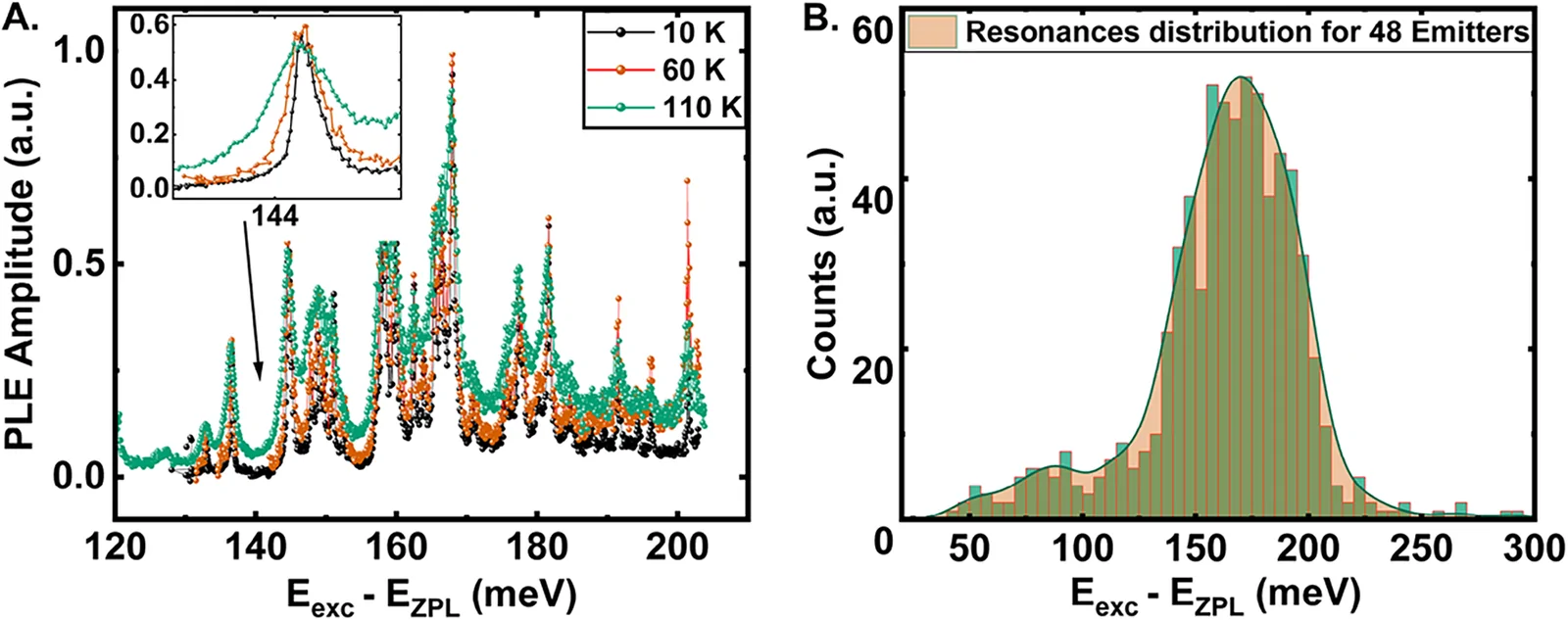
(A) Resonance energies shown for an emitter H at different temperatures namely, 10, 60, and 110 K. The inset shows a zoomed-in view around 144 meV energy difference. (B) Distribution of resonance peak energies calculated as difference of excitation energy and ZPL energy for 48 different emitters.

To determine whether the observed resonances are specific to individual emitters, we performed a systematic study of multiple emitters. Not all emitters exhibited resonances, and among those that did, the resonance energies varied among emitters. In this experiment, we show the data for 48 different emitters displaying statistically significant resonance peaks. The energies of the resonance peaks were plotted on a relative energy scale, and a histogram was constructed with a bin width of 5 meV, as shown in Figure 4B. Resonances were observed within the 50–300 meV excitation range relative to the ZPL position, with an apparent higher density of states around 170 meV. So far, in our measurements, no correlation was observed between the ZPL energies of the emitters and the presence or energies of the resonances in our measurements.

Furthermore, we have investigated multiple emitters across different samples and, to date, have not observed any irreversible modification or disappearance (“bleaching”) of the resonance patterns. This indicates that the resonances are intrinsically linked to the emitter and persist as long as the emitter remains optically active.

### Low-Energy Peaks

Finally, we test whether the excitation resonances have corresponding features on the low-energy side of the ZPL, as expected if the absorption process involves localized vibrations coupled to the electronic transition. One such exemplary PLE map, for emitter I, is shown in Figure 5A. The map highlights a few important features. The ZPL, as marked, is located at about 1.945 eV. The excitation energy was swept from 2.09 to 2.18 eV. It can be seen that the ZPL passes through several resonances in this scanning range. Also on the lower detection energy, three faint lines marked as E1, E2, and E3 can be seen. Their positions mirror those of the absorption peaks. The lines are dim in nature, but the intensity of these lines is positively correlated with the intensity of the ZPL. The Cr^3+^ lines in sapphire are also marked at a detection energy of about 1.769 eV. During all PLE measurements, the excitation power was monitored, and the extracted PLE amplitudes were normalized by the measured laser power. Power-dependent measurements confirmed that the emitters were operated in the linear excitation regime, while the faint background fluctuations visible in the PLE maps may arise from background channels that may not be in the same linear regime. Figure 5B,C shows the zoomed-in view of the lower-energy line E2 and ZPL of the emitter. The zoomed-in area is marked with a white rectangle in Figure 5A. As also evident from here, the intensity pattern of the emission line E2 and ZPL is strongly correlated.

**Figure 5:**
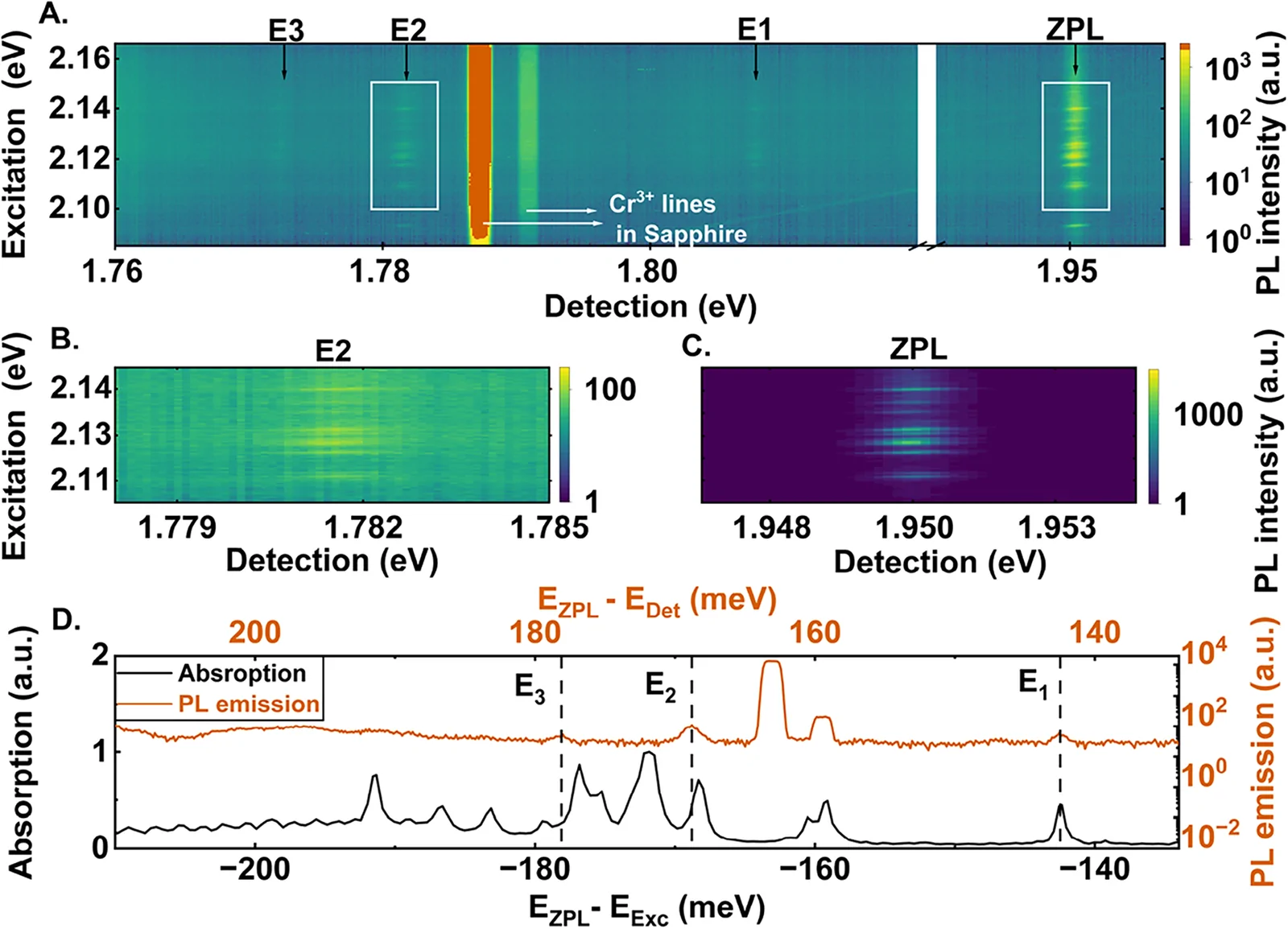
(A) PLE map of emitter I showed in the panel. The ZPL and Cr^3+^ lines from the sapphire has been marked accordingly (orange color marks the saturation region). The lines E1, E2, and E3 are marked in the PLE map, which correspond to low-energy peaks. When the resonances cross through ZPL, emission lines at E1, E2, and E3 lines are also seen to get brighter. (B, C) Zoomed-in view of the lines E2 and ZPL (marked by white rectangle on map) have been presented. (D) The bottom panel shows the PLE amplitude of the ZPL in the black color with respect to the ZPL energy. The cross-section of the PLE map is also plotted in the orange color. As can be seen, the lines E1, E2, and E3 are mirrored at the same energy distance on the lower-energy side of the ZPL.

The graph in Figure 5D shows the PLE amplitude of the ZPL in the black color. As can be seen, the resonances are present in the range of 140–200 meV away from the ZPL position. The orange line shows a cross-section through the PLE map at 2.12 eV excitation energy plotted as the PL emission. The Cr^3+^ lines from the sapphire substrate are also visible in the spectra. The PL emission lines occur at energies corresponding to the resonances observed in absorption, although a perfect spectral overlap was not observed. Additionally, we note that not all absorption resonances have corresponding features in the PL emission.

### Discussion

The central question raised by these observations is which microscopic mechanism can account simultaneously for the narrow line widths, polarization selectivity, emitter-dependent resonance patterns, and characteristic energy scale around 170 meV. Although the complete microscopic origin of the resonances is not yet fully established, our results offer relevant clues and enable a discussion of their possible origins.

Bulk phonons are one of the candidates to explain the observed resonances. However, their energies in GaN occur at significantly lower-energy scales (approximately 70 meV for the *E*_2_ (high) mode and 92 meV for the *E*_1_(LO) mode), indicating that the observed resonances cannot be attributed to coupling with the principal bulk phonons. The 140–200 meV energy range corresponds well with the multiphonon absorption.^23^ However, the relative resonance energies varies between emitters and no universal pattern could be attributed to a combination of two well-defined (at Γ-point) phonons.

Several studies indicate that donor–acceptor pairs (DAPs) are responsible for single-photon emission in hexagonal boron nitride (hBN). One study explicitly states that most single-photon emitters in hBN can be well explained by donor–acceptor pairs, with their wavelength fingerprints matching the experimentally observed photoluminescence spectrum.^24^ Potentially, a similar mechanism could be responsible for the multiplicity of the emission energies of the GaN single-photon defect centers. However, the donor–acceptor pair band transitions in GaN are situated at around 3 eV energy range,^25^ so well above the energy of the ZPL observed in our experiments. With rising temperature, the ZPL observed in our experiments monotonically red shift by around 3 meV (from 4 to 180 K), closely resembling the behavior described in ref 26. The energy difference between the observed resonances and ZPL is constant with temperature, as observed in Figure 4A. The change of energy of the donor–acceptor pair with temperature can be nonmonotonic^27^ and shift in even up to 100 meV scale.^28^ Such observations point toward associating the observed resonances with localized vibrational modes (LVM) rather than phenomena related to DAP.

We hypothesize that the resonances may be associated with localized vibrational modes (LVMs). The defects responsible for single-photon emission in the visible range in GaN are plausibly associated with complexes of substitutional impurities and vacancies.^15,20^ In particular, theoretical calculations for the N_Ga_V_N_^0^ defect complex suggest that localized vibrational levels may exist at energies corresponding to those of the observed resonances.^20^ The N_Ga_V_N_ complex may therefore represent a possible electronic core of the emitter rather than its complete microscopic structure. More generally, the optically active center may be a multicomponent vacancy–impurity complex, in which the electronic transition is primarily associated with such a core, while the observed resonances originate from localized modes involving additional atoms.

The large energy of the LVM would point toward the light additional atoms, such as C, H or N, which are abundant in MOVPE-grown GaN samples. Carbon-containing defects and complexes in GaN are known to exhibit distinct localized vibrational fingerprints,^23^ while hydrogen can bind to vacancies and impurities and substantially modify both their electronic structure and local vibrational spectrum.^17^ Nitrogen-rich configurations containing an N–N dimer-like unit, including nitrogen split-interstitial structures, constitute another possible realization of a light-atom-containing complex.^29,30^ An N–N unit with an effective bond order close to two may support a stretching vibration near 180 meV, close to the maximum of the resonance-energy distribution observed in our measurements.^31^

We note that multiple peaks cannot be overtones of a single LVM, which is in agreement with the small Huang–Rhys factor observed for these defects.^5^ A plausible explanation would be the presence of multiple local modes, also supported by their polarization selectivity, which could be associated with the geometry of defects. The measured line width of the resonances (0.5 meV) and the occasional presence of emission lines on the lower-energy side of the ZPL, mirroring the absorption peaks as shown in Figure 5, further support the association of the observed resonances with LVMs.

Within this picture, the broad distribution of ZPL energies would mainly reflect variations in the electronic structure of the defect core caused by its composition, charge state, strain, and the local electrostatic environment. In contrast, variations in the resonance detunings would reflect differences in the local vibrational spectrum arising from the composition, bonding, and geometry of the complete defect complexes. Differences in local symmetry and defect configuration could, in turn, explain the different polarization characteristics. The electronic and vibrational properties may therefore vary partly independently, naturally accounting for the absence of a simple correlation between the ZPL energy and the observed resonance patterns.

A complete understanding of the origin of these resonances and their relationship to specific defect configurations requires further theoretical investigation. We hope that our experimental evidence will allow us to restrict the theoretical search for specific defect centers.

## Conclusions

In this work, we report an experimental observation of resonances in photoluminescence excitation of GaN single-photon emitters operating in the visible spectral range. The resonances are likely related to the phonon-assisted excitation. We demonstrate that quasi-resonant excitation through these states can enhance the zero-phonon line (ZPL) emission of individual emitters by up to an order of magnitude. The resonances can be selectively addressed via the polarization of the excitation light, while the emission dipole orientation of the emitter remained unchanged. Statistical analysis indicates that these resonances occur most frequently around 170 meV. We attribute the origin of the resonances to coupling between the emitter and localized phonon modes, which gives rise to sharp spectral features in the photoluminescence excitation (PLE) spectra. Consequently, these resonant states provide a quasi-resonant excitation pathway, enabling an increased emission into the ZPL. Our findings offer a pathway to enhance the brightness of GaN single-photon emitters and provide valuable insights into the energy-level structure of the underlying defects, facilitating their integration into photonic platforms.

## Experimental Setup

The GaN sample studied in this work consists of two GaN layers (1.5 μm buffer layer and 3.5 μm of controlled carbon concentration layer) grown on a sapphire substrate using an Aixtron CCS-FT 3 × 2 MOCVD system. The carbon concentration in the sample was 2.3 × 10^17^/cm^3^. To estimate the emitter density, we used a room-temperature confocal scanning microscopy setup where the sample was illuminated with a 532 nm green laser diode, and the signal was collected on an avalanche photodiode after spectral filtering of the detected signal. To confirm the single-photon nature of the emitters, we used two PerkinElmer fast photodiodes mounted at the exit of two 700 mm long spectrometers in Czerny-Turner configuration, equipped with gratings up to 2400 lines/mm and a CCD camera featuring pixel size of 16 μm. The excitation photon energy was continuously monitored using a calibrated wavemeter, providing high-precision determination of the excitation energy. The emission energies, including the zero-phonon line (ZPL) and lower-energy spectral features, were measured using the spectrometer that was calibrated using known reference lines prior to the measurements.

For correlation measurements, emission signal from selected emitters was equally divided using a 50:50 nonpolarizing beam splitter and directed to two PerkinElmer single-photon avalanche photodiodes (SPADs) positioned at the output ports of successive stages of the *TriVista* spectrometer. Second-order photon autocorrelation measurements were performed by using a PicoQuant *HydraHarp* time-correlated single-photon counting module.

The high refractive index of GaN decreases the setup’s collection efficiency. For experiments requiring high number of photon counts, we employed a cryo-compatible immersion objective^21^ in contact with the GaN sample surface to address this issue. This approach also allowed us to significantly minimize chromatic aberrations during photoluminescence excitation studies. The sample and excitation objective were mounted inside an Oxford Spectromag helium-bath cryostat. For broadband excitation spectroscopy, a supercontinuum source was employed to cover the energy range from 2.40 to 1.90 eV (520–650 nm). For high-resolution excitation scans, a tunable dye laser was used, providing continuous coverage from 2.03 to 2.17 eV (570–610 nm). The detection channel was spectrally filtered using interference filters to isolate emission in the range of 1.77–2.06 eV (600–700 nm). A linear polarizer and a rotatable half-wave plate were incorporated into both the excitation and detection optical paths to enable complete characterization of the emitters’ absorption and emission polarization properties.

## Supplementary Material



## Data Availability

The data that support the findings of this study are available from the corresponding author upon reasonable request.

## Notes

Additional details about the correlation measurements, power dependence of emitters, and polarization-resolved photoluminescence excitation spectroscopy (PDF)
