# Supplementary material for: Efficient Quasi-Resonant, Polarization-Selective Excitation of GaN Quantum Emitters
Source: ACS Photonics. 2026 Oct 15;13(21):6579–87. doi: 10.1021/acsphotonics.6c02170 (PMC13742290; doi:10.1021/acsphotonics.6c02170)
Supplement: Supplementary file 1 [file ph6c02170_si_001.pdf]

SUPPLEMENTARY INFORMATION

Efficient Quasi-Resonant, Polarization-Selective Excitation of GaN Quantum Emitters

Nilesh Dalla, † Paweł Kulboka, † Michał Kobecki, † Jan Misiak, † Paweł Prystawko, ‡ Tomasz Kazimierczuk, † Piotr Kossacki, † Henryk Turski, ‡ and Tomasz Jakubczyk* †*

† University of Warsaw, Faculty of Physics, Institute of Experimental Physics, Ludwika Pasteura 5, Warsaw, 02-093, Poland

‡ Institute of High Pressure Physics "Unipress", Polish Academy of Sciences, Sokołowska 29/37, Warsaw, 01-142, Poland

E-mail: n.dalla@uw.edu.pl; tomasz.jakubczyk@fuw.edu.pl

1. Single Photon Nature:

We have performed autocorrelation measurements using HBT scheme to verify the single photon nature of these defects. To measure the g(2) we used a 585nm excitation using a rhodamine tunable laser. In the detection the signal was filtered from the laser, split by a 50:50 beam splitter and directed to photodiodes after passing through the monochromators. The ZPL of 2 such emitters as well as the coincidence counts are provided below.

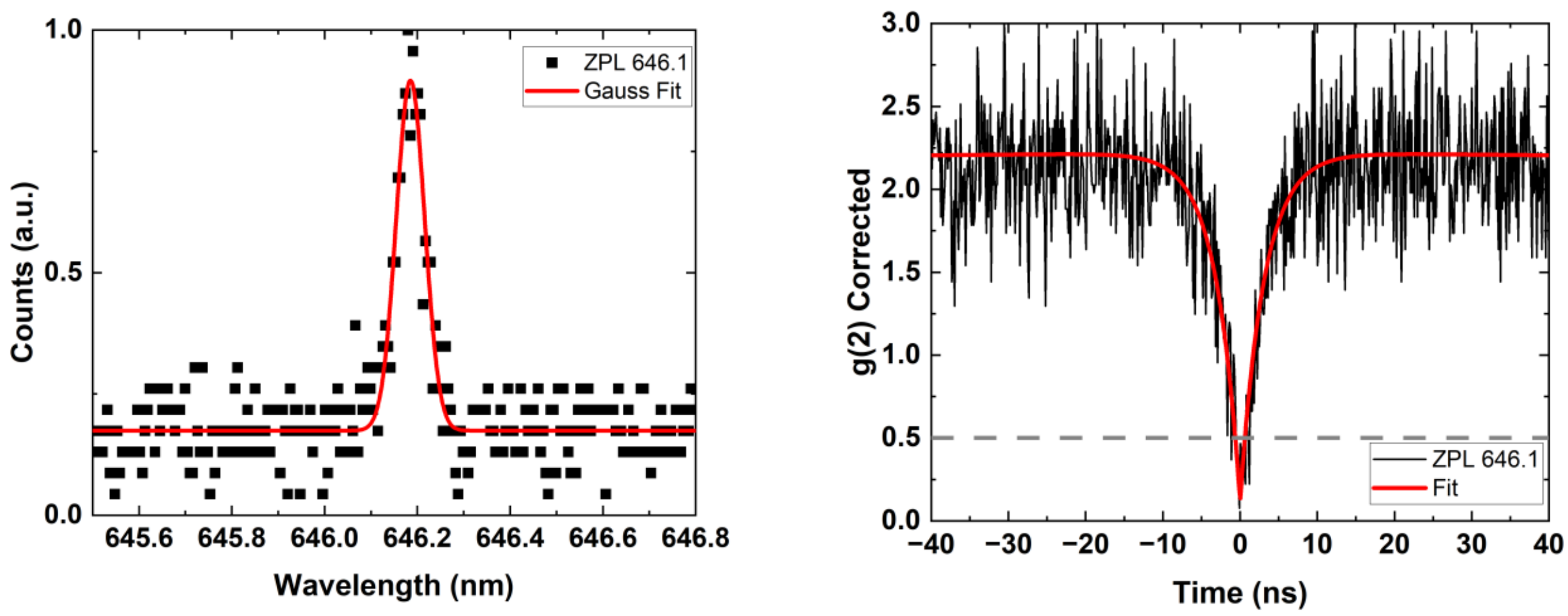

A) B)

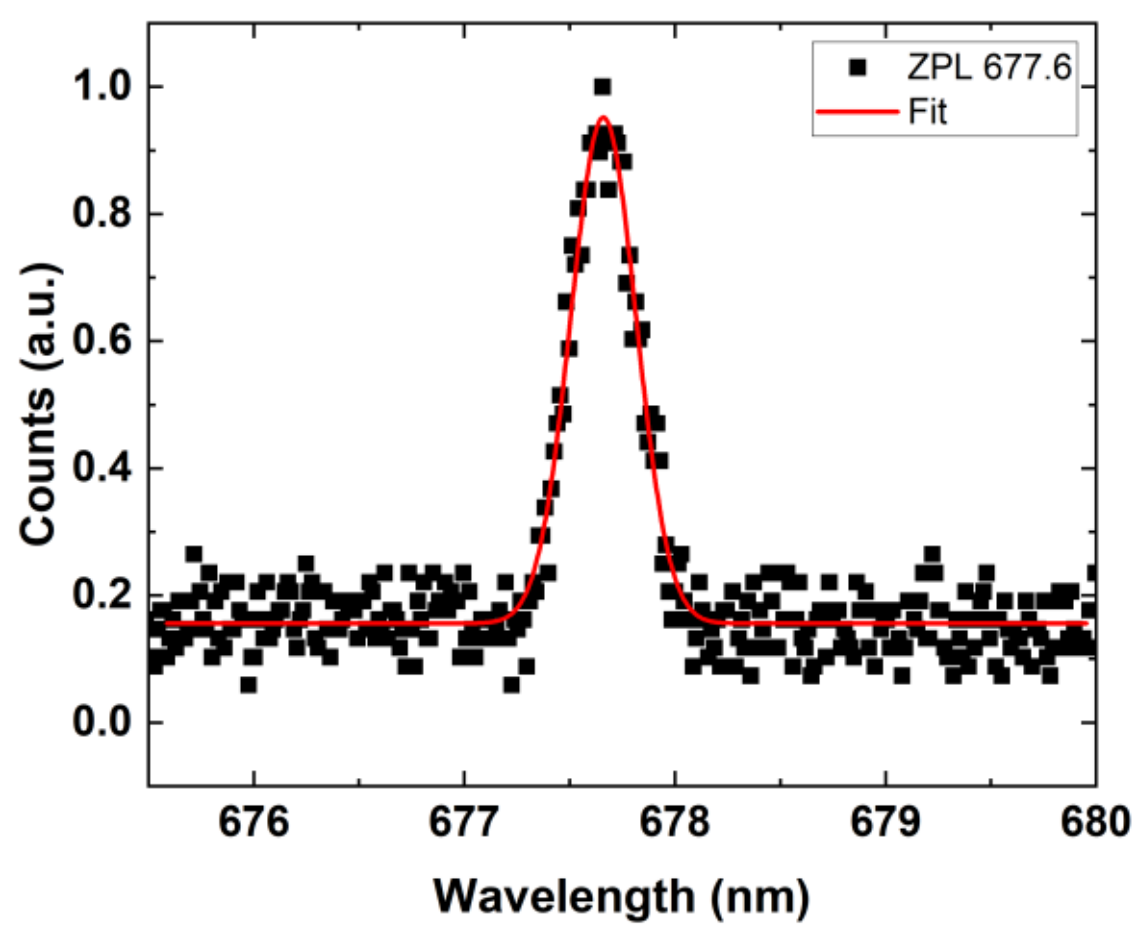

C)

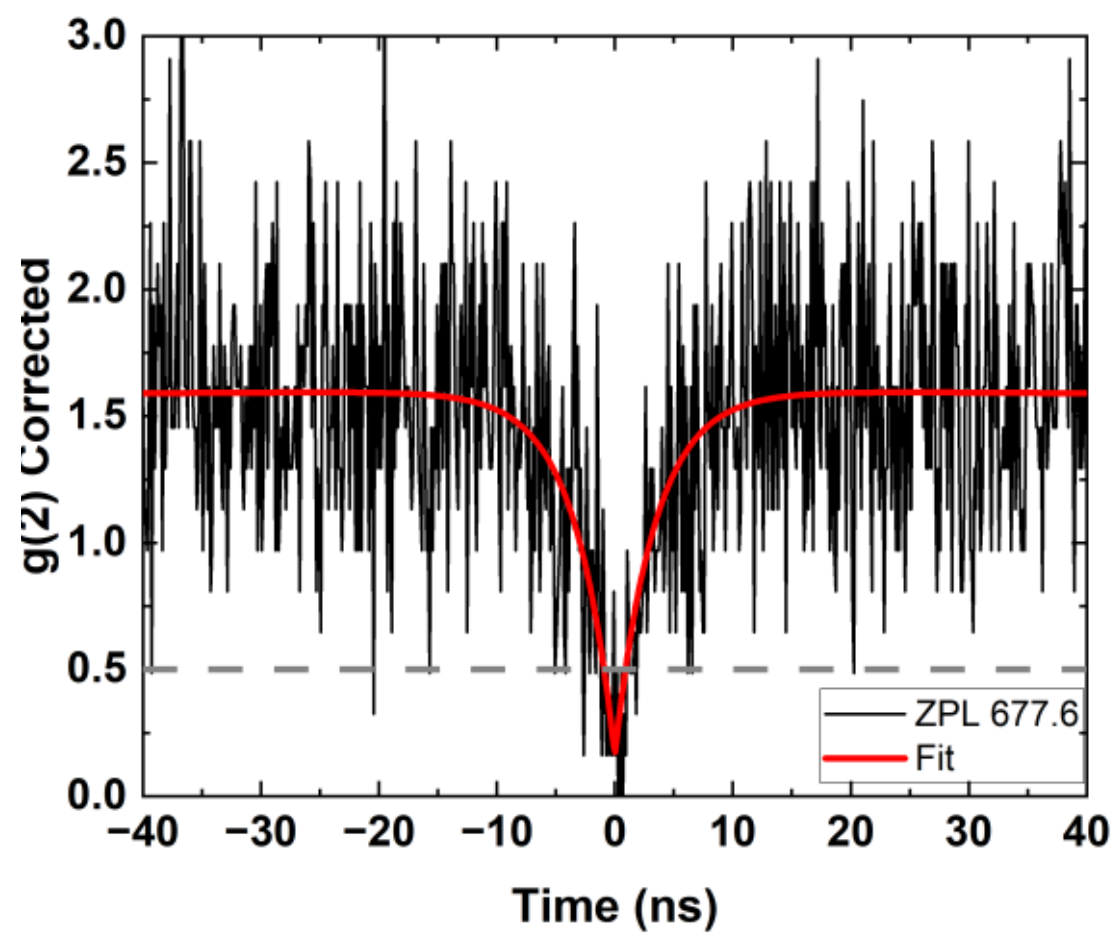

D)

Figure S1: *Photoluminescence spectra of two emitters at ZPL 646.1 nm (emitter A0) and ZPL 677.6 nm (emitter A1), excited by 585 nm laser at 10 K temperature, shown in figure A) and C) respectively. The corresponding correlation function after normalization and background correction for emitters has been plot in the figures B) and D) respectively.*

The coincidence counts were normalized for the experimental parameters the raw coincidence counts, Cn, were normalized with respect to the acquisition time, T the temporal bin width, w, and the individual detector count rates, N1 and N2, as given by Eq. 1.

$$C_n = \frac{C}{N_1 N_2 w T} \tag{1}$$

$$g(2)_{corrected} = \frac{(C_n - (1 - S_n^2))}{S_n^2} \tag{2}$$

The resulting normalized counts, Cn, were subsequently corrected for background contributions according to Eq. 2, where Sn denotes the measured signal-to-noise ratio of the emitter line measured on the spectrometer, and g(2) corrected represents the background-corrected second-order correlation function. The normalization procedure was performed so that at longer time delays the g(2) approaches unity and then fitted using the expression in (3) for a 3-level system [1].

$$g(2) = \left(1 - a.\exp\left(-\frac{|t|}{\tau^{fast}}\right)\right)\left(1 + b.\exp\left(-\frac{|t|}{\tau^{slow}}\right)\right) \tag{3}$$

ZPL (nm)	FWHM (nm)	ZPL (eV)	FWHM (meV)	g2(0)	τ^{fast} (ns)
646.1 ± 0.1	0.14 ± 0.02	1.91	0.41	0.11	3.08 ± 0.16
677.6 ± 0.2	0.36 ± 0.06	1.82	0.97	0.15	3.37 ± 0.39

Table S1: Measured ZPL positions, FWHM, g2(0) and τ^{fast} for emitters shown in figure S1A and S1C.

Figure S1A and S1C shows different emitters with distinct ZPL energies And S1B and S1D shows their corresponding correlation counts. As can be seen from figures S1B and S1D, the correlation function goes well below 0.5 at zero delay, hence signifying the single photon emission behavior for both the emitters. The table above summarizes the parameters such as ZPL, FWHM, g2(o) and T^{fast} for both studied emitters. The excitation power was kept at 1 mW for both the emitters.

G(2) at different Excitation powers for the emitter with the ZPL at 646.1 nm (emitter A0):

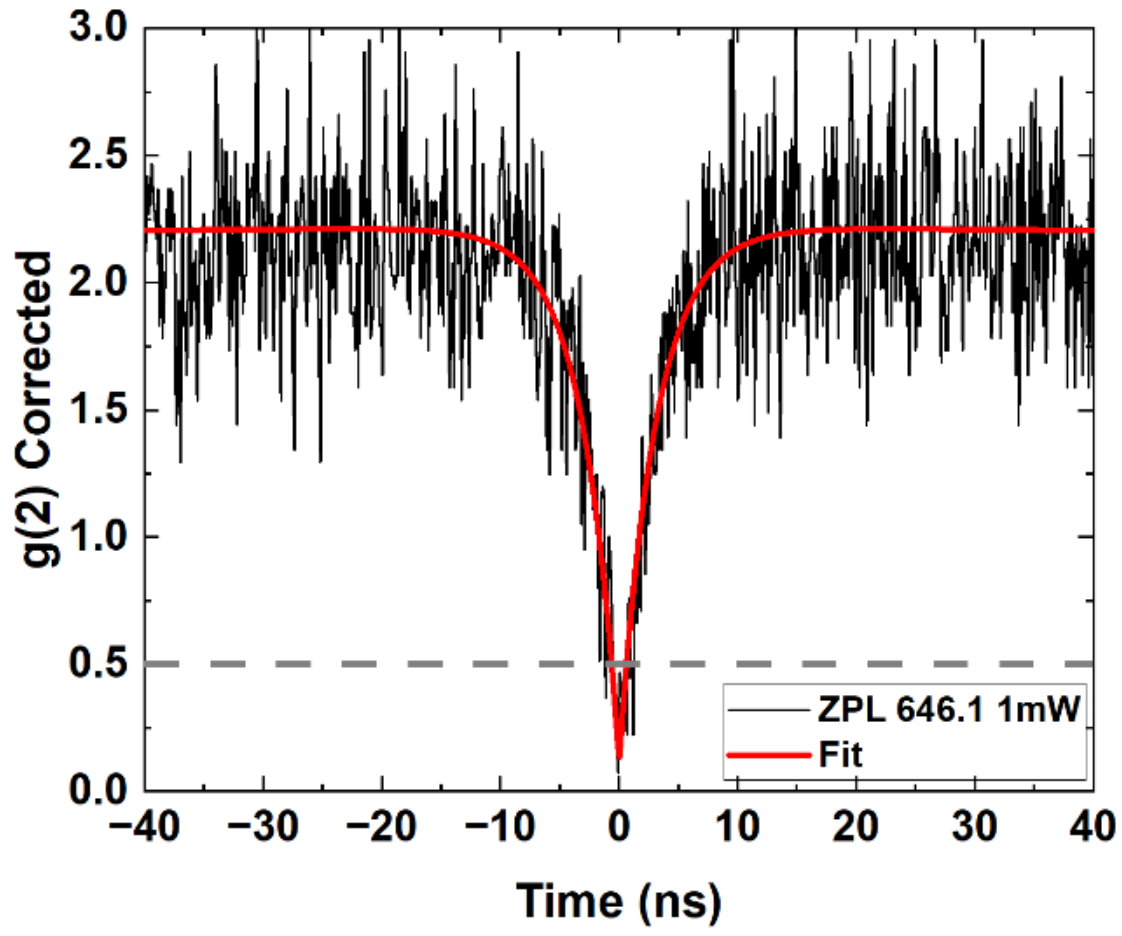

A)

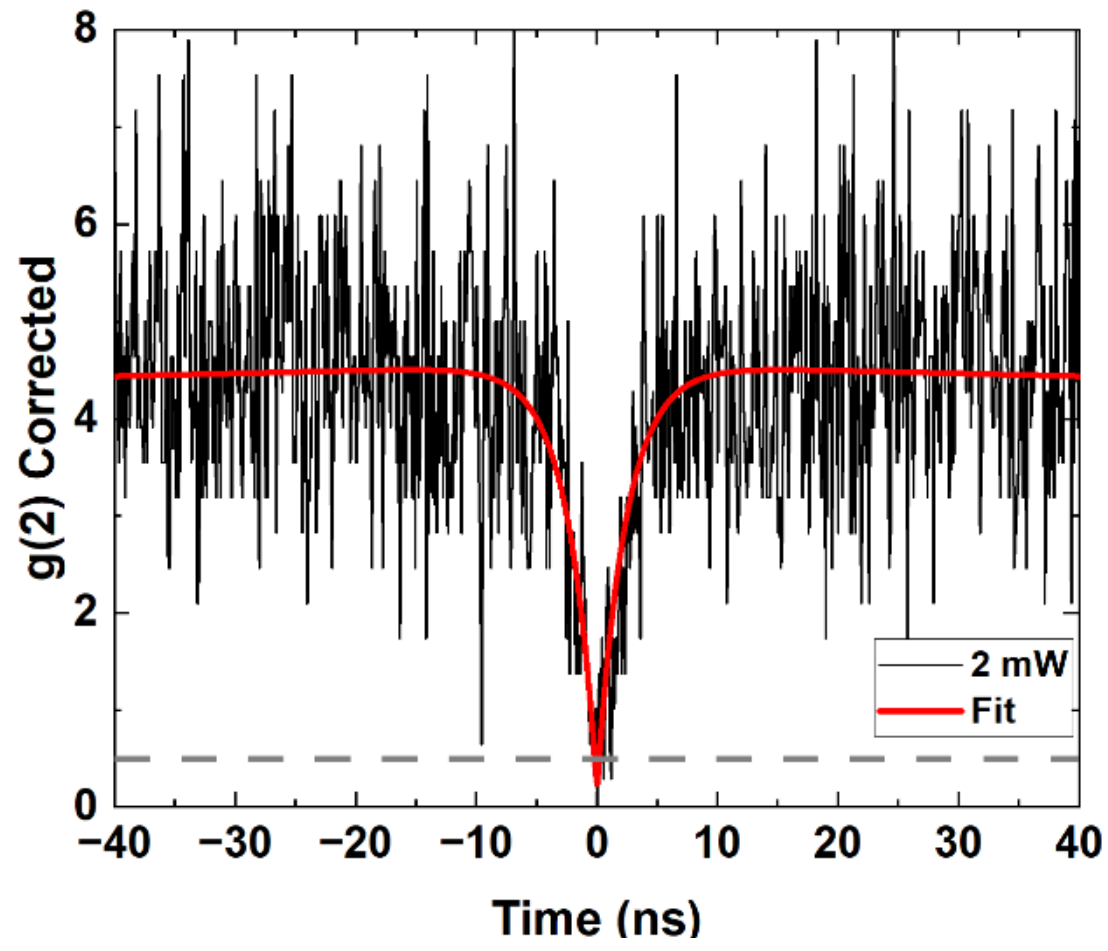

B)

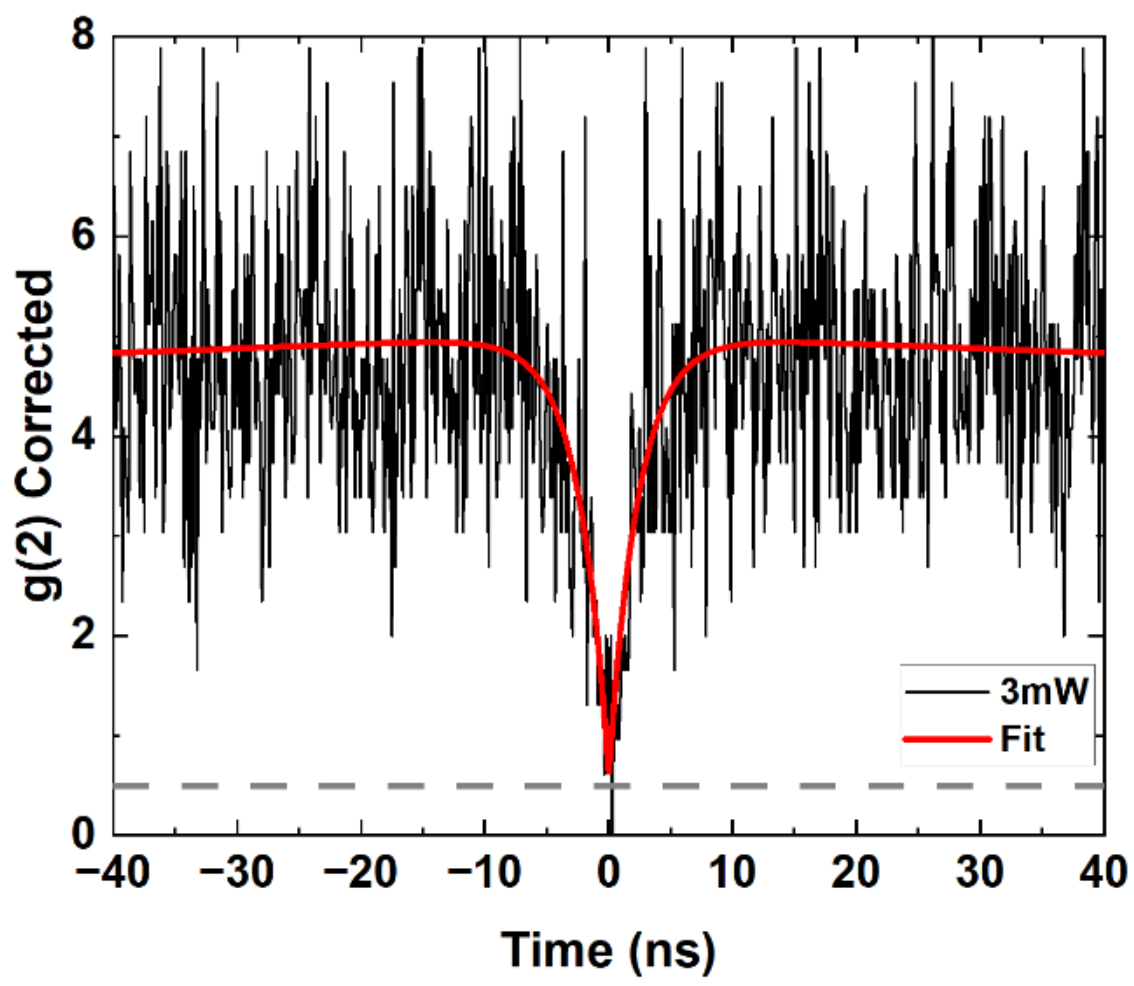

C)

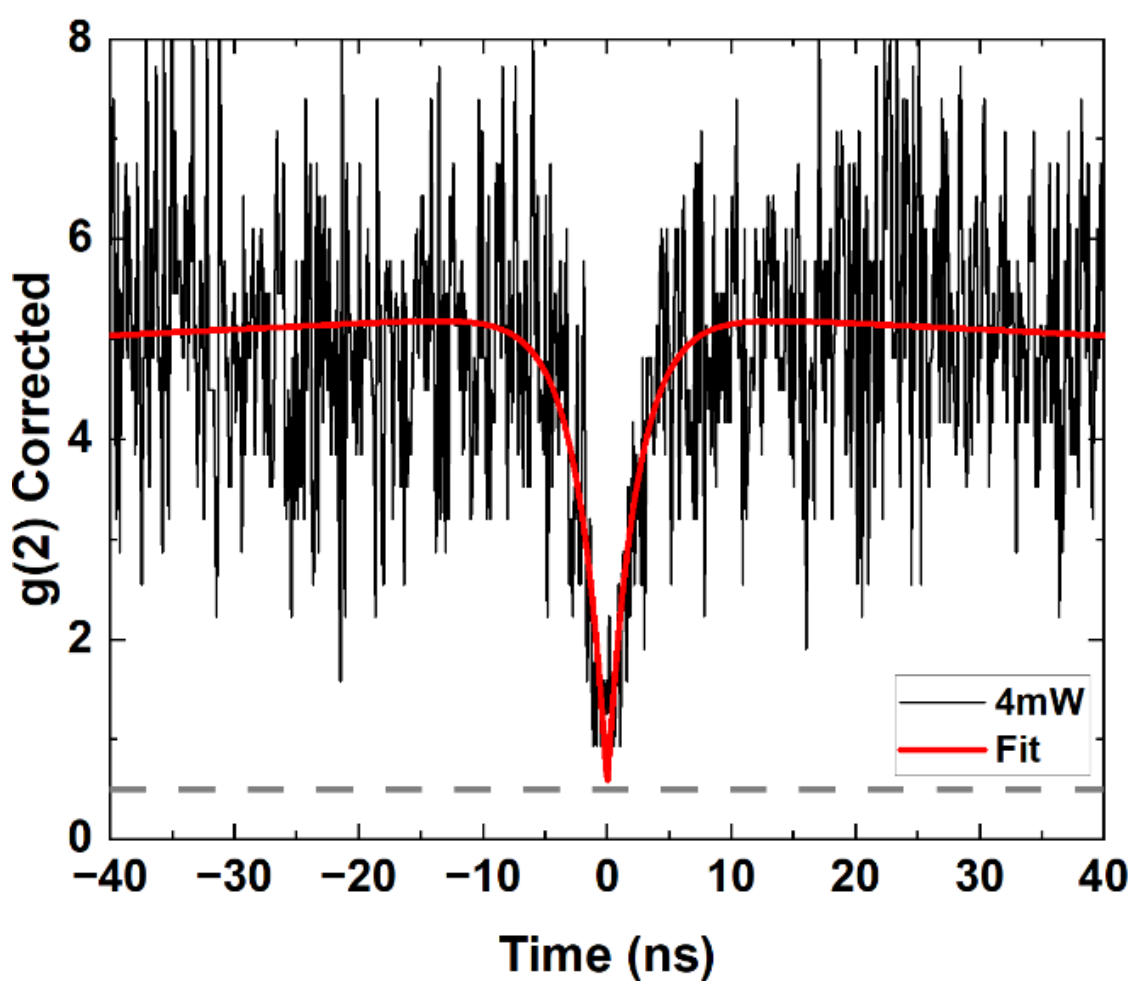

D)

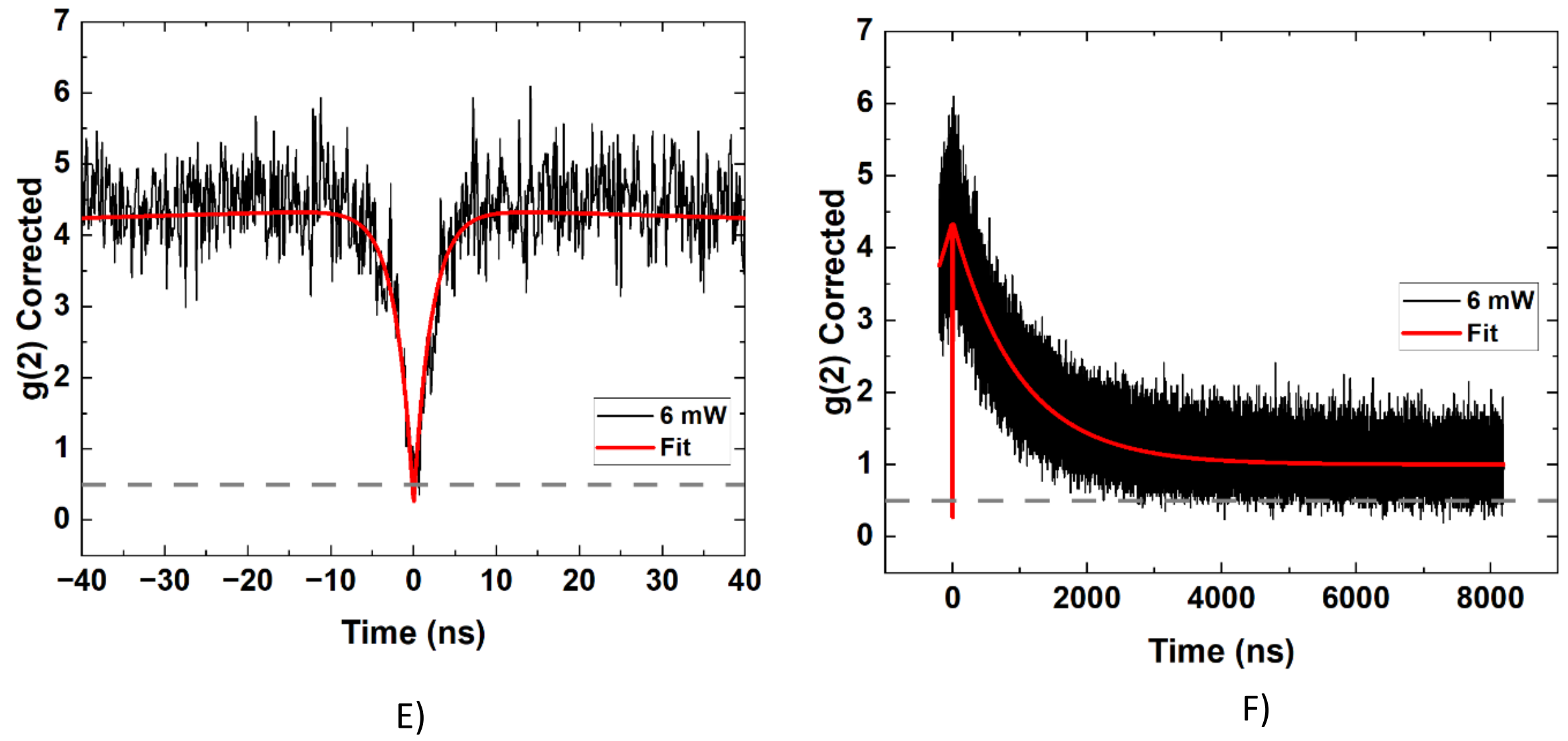

Figure S2: A)-E) *Background corrected g(2) at different excitation powers for the emitter at ZPL 646.1 nm (emitter A0) from excitation power ranging from 1-6 mW. All the plots have been normalized for as shown in the figure above. F) Long range g(2) plot for 6 mW excitation power.*

On the figure S2A-E, we present the background corrected incident counts for emitter A0 for different excitation powers (1-6 mW). The excitation energy was kept constant throughout the scans at 585 nm. As can be seen from the plots, as the excitation power is increased the g2(0) level rises. Figure S2F, displays the long range correlation function for 6mW excitation power.

2. Excitation Power Dependence:

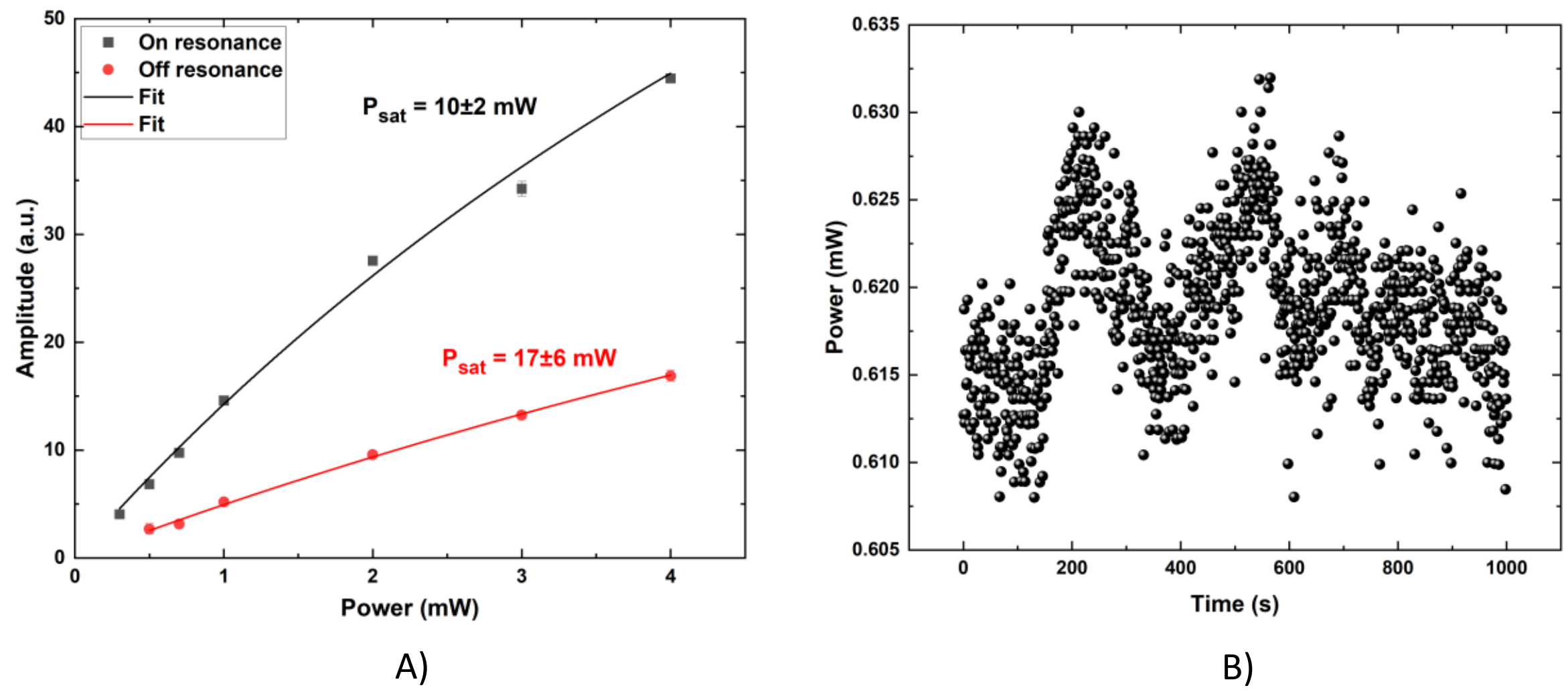

Figure S3: A) *Extracted fit amplitude from Photoluminescence spectra of an emitter for different excitation energies, namely on and off resonance energy. B) Laser power plotted in time showcasing the stability of the laser power.*

In our experiments we have used a solid immersion objective to increase photoluminescence collection efficiency. Please note that the actual power density at the emitter depends on many factors related to the construction of the used immersion objective including position of the emitter with respect to the axis of objective and the size of the beam entering the pupil of the objective.

Only a portion up to 50% is focused on the sample (see reference [2] for the design of the objective), while the rest is back reflected on the optical axis.

To examine closely the power dependence of emitters, we have also measured PL spectra at different excitation powers and at different excitation energies, namely on and off resonance shown in figure S3A. By fitting saturation curves to both On and Off resonance (shown with a solid line labelled as fit) we extract saturation powers as 10 mW and 17 mW respectively. Figure S3B shows the power fluctuations observed in time. We see less than 1 percent RMS fluctuations and about 4 percent peak to peak variation for an interval of 1000 secs. Since we mainly work in the linear regime, we are normalizing each collected spectra with the laser power to eliminate any fluctuations coming from laser power. In our experiments, we don't see any direct correlation between excitation power and quantities such as FWHM or ZPL energy.

3. Polarization resolved PLE:

The polarization resolved PLE spectra was collected for 44 different emitters. The excitation polarization was tuned with respect to the GaN high symmetry m-axis direction. Each collected PLE spectra at every excitation angle was power normalized.

Total emitters measured	Emitters lost mid scans	Emitters with resonances among measured successfully	Emitters without any measurable resonances among measured successfully	Emitters with resonances with same phase for all peaks (Group 1)	Emitters with resonances with different phase for 1 or more peaks (Group 2)
**44**	11 %	79%	21%	82%	18%

Table S2: *Statistical summary of all the measured emitters.*

Here we provide a summary of all different emitters. Table S2 shows the statistical measurements of the emitters that were measured, among which 11 percent emitters were lost during measurements. Among those measured 79% showed resonances and among with resonances measured about 82% belonged to the Group 1 emitters and about 18% belonged to the group 2 emitters. Group 1 and group 2 emitters are described in detail in the figure 3 in main text.

Below we present the collected distribution for all measured 44 emitters.

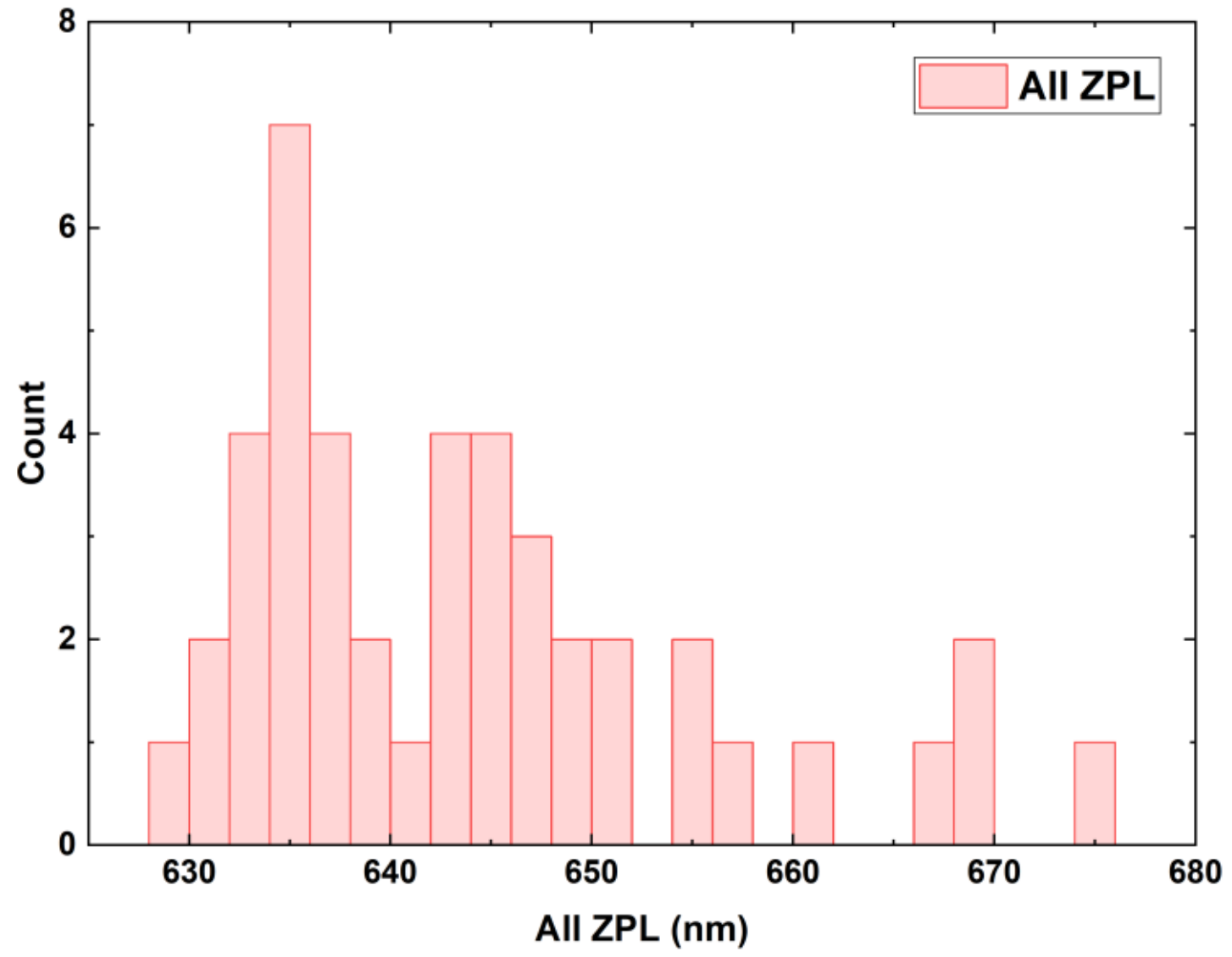

Figure S4: *Extracted ZPL position of all the measured emitters.*

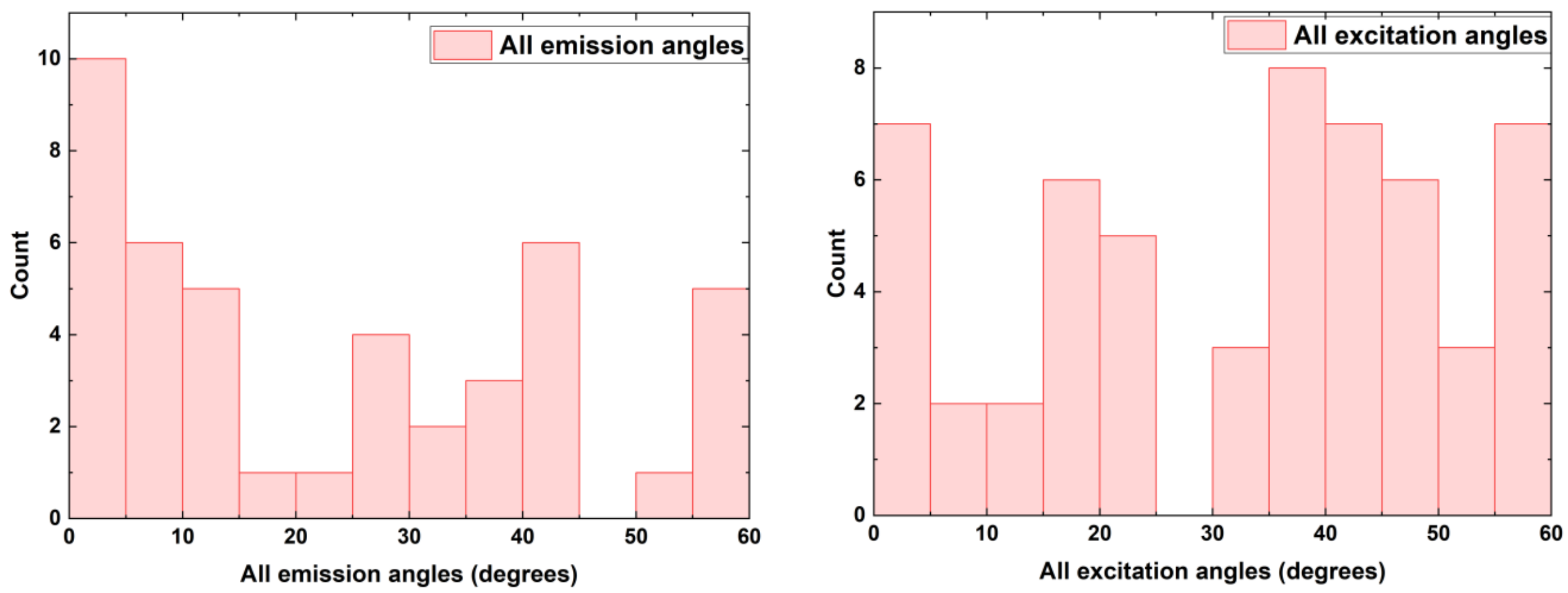

A) B)

Figure S5: A) *Measured orientation of the emission dipole angles of all the emitters. B) Measured orientation of the absorption/excitation dipole angles of all the emitters. All the angles are measured with respect to the GaN in plane m axis.*

Figure S4 shows the overall ZPL energy statistical analysis of the measured emitters. Each emitter was fitted with a gaussian peak to extract the peak position. Figure S5A and S5B shows the emission and absorption dipole angle orientation of all the measured emitters. The ZPL was fitted with a gaussian peak to extract the PL amplitude and then fitted with a cosine function to extract dipole angle orientation for emission and excitation. All displayed angles have been shown after mod 60 operation because of in-plane symmetry of GaN crystal.

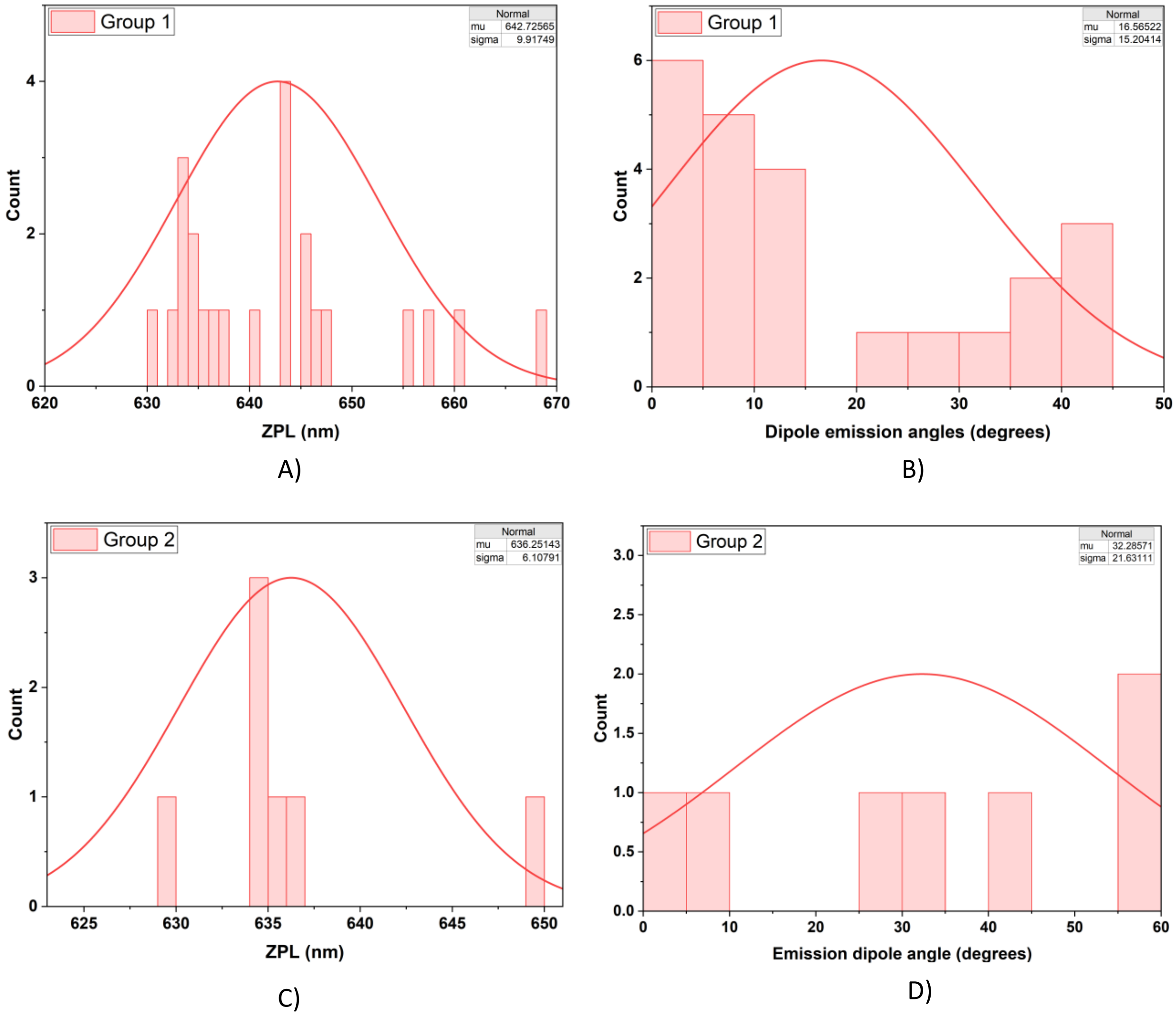

Figure S6: A) *Distribution of ZPL emission energy of group 1 emitters. B) Distribution of dipole emission angles of group 1 emitters. C) Distribution of ZPL emission energy of group 2 emitters. D) Distribution of dipole emission angles of group 2 emitters.*

Figure S6A shows the ZPL emission energies of group 1 emitters. And figure S6B shows the detection dipole angles with respect to the GaN m-axis direction for group 1 emitters. Similarly figure S6C shows the ZPL emission energies of group 2 emitters. And figure S6D shows the detection dipole angles with respect to the GaN m-axis direction for group 2 emitters. So far from our measurements, we do not see a clear correlation between group 1 and 2 emitters on parameters such as ZPL emission energy or emission dipole angles.

References:

1. Sallen, G., Tribu, A., Aichele, T. *et al.* Sub nanosecond spectral diffusion measurement using photon correlation. *Nature Photon* **4**, 696–699 (2010).
2. Jasny, J. *et al.* Fluorescence microscopy in superfluid helium: Single molecule imaging. *Review of Scientific Instruments* **67**, 1425–1430 (1996).
